# The Role of Vitamin D Deficiency in Children With Recurrent Wheezing—Clinical Significance

**DOI:** 10.3389/fped.2020.00344

**Published:** 2020-06-30

**Authors:** Gavriela Feketea, Corina I. Bocsan, Luminita Aurelia Stanciu, Anca Dana Buzoianu, Mihnea Tudor Zdrenghea

**Affiliations:** ^1^Department of Hematology, “Iuliu Hatieganu” University of Medicine and Pharmacy, Cluj-Napoca, Romania; ^2^Department of Pediatrics, Pediatric Allergy Outpatient Clinic, “Karamandaneio” Children Hospital, Patras, Greece; ^3^Department of Pharmacology, Toxicology and Clinical Pharmacology, “Iuliu Hatieganu” University of Medicine and Pharmacy, Cluj-Napoca, Romania; ^4^Airways Disease Infection Section, National Heart and Lung Institute, Imperial College London, London, United Kingdom; ^5^Department of Hematology, Ion Chiricuta Oncology Institute, Cluj-Napoca, Romania

**Keywords:** vitamin D, 25(OH)D, vitamin D receptor (VDR), recurrent wheezing, asthma, respiratory allergies

## Abstract

Recurrent wheezing (RW) in infancy is one of the most frequent reasons for parents to consult health care providers and creates a significant global burden. Clinical course of RW is difficult to predict, also which infants will progress to asthma, since no valid biomarkers have been established. Identification of those infants with RW who are at risk of further recurrences and/or severe acute respiratory tract infection (ARTI) could help pediatricians to improve their therapeutic decisions. Increasing research interest is focused on the extra-skeletal actions of vitamin D (VD) and the clinical impact of VD insufficiency/deficiency. As VD deficiency could be a risk factor for causing RW in children, measurement of their serum level of 25-hydroxycholecalciferol [25(OH)D] is recommended. In the case of deficiency, VD administration is recommended in age-appropriate doses for at least 6 weeks, until achievement of normal blood 25(OH)D level, followed by supplementation as long as exposure to sun is inadequate. Higher doses of VD given in an attempt to prevent asthma development appear to be of no additional benefit. In children with severe ARTI, VD level is recommended to be assess.

## Key Points—Questions

Could vitamin D status be a biomarker for risk of ARTI in children with recurrent wheezing?

Does vitamin D supplementation affect the incidence and clinical course of ARTI?

Does vitamin D supplementation modify development of respiratory allergies (asthma)?

## Meaning

– Children with recurrent wheezing may be vitamin D deficient, and their serum level of 25(OH)D would be useful for identifying those children who would benefit from vitamin D supplementation.
– Vitamin D supplementation may reduce the risk of respiratory infection and asthma exacerbation in some clinical contexts. In the case of deficiency, vitamin D should be administered in daily dose depending on age, for at least 6 weeks, until achievement of a normal serum 25(OH)D level, followed by supplementation when sun exposure is inadequate.
– In deficient children, higher doses of vitamin D appear to provide no extra benefit in modifying the clinical course of ARTIs or preventing asthma.

## Introduction

Vitamin D (VD) research has been focused increasingly on its extra-skeletal actions and its possible role in immune system modulation, and on the clinical impact of VD insufficiency. Viral lower respiratory tract infection, acute viral bronchitis, acute bronchiolitis, viral pneumonia, viral wheeze, recurrent/transient/multi-trigger wheezing, and viral induced exacerbation of asthma are only a few of the vast range of terms used as diagnostic labels for respiratory illnesses cause by respiratory viruses in infancy and early childhood (1). Recurrent wheezing (RW) in infancy is one of the most frequent reasons for parents to consult health care providers and constitutes a huge global burden. This review analyzes the current evidence for the relationship between VD status and RW in infancy and childhood, including potential progress to asthma, and the effects of VD supplementation.

## Recurrent Wheezing

Many different conditions can produce “wheezing,” which is a musical sound caused by the passage of air through narrow respiratory tract airways, but this airway narrowing is caused most often by acute respiratory tract infection (ARTI) (2). RW in children aged ≤5 years is a heterogeneous condition, typically associated with recurrent upper respiratory tract infections (URTIs). As each patient may have 6–8 episodes of URTI per year, the question of whether a wheezing episode is an initial or a recurrent clinical event constitutes a challenge to the clinician, but RW is generally defined as 2 or more episodes of reported wheezing since birth. Wheezing phenotypes proposed by the European Respiratory Society (ERS) Task Force in 2008 differ, based on the criteria used for classification: episodic viral or multiple-trigger wheeze, according to symptom-based classification; transient, persistent and late-onset wheeze, according to time trend-based classification (3). Theoretically, this approach enables individual therapeutic decisions to be made based on the temporal pattern of symptoms (4). In clinical practice, so many infants and young children present wheezing with viral infections that early allocation to one of these phenotypes is unrealistic (5). Identification of those infants with RW at risk for future recurrence and/or severe evolution could help pediatricians to optimize their therapeutic decisions.

## Vitamin D

### Sources and Metabolism

VD in the human organism comes from exposure to sunlight and from food and supplements. Ultraviolet B radiation converts 7-dehydrocholesterol to previtamin D_3_ and subsequently to vitamin D_3_ (6). Foods provide vitamins D_2_ and D_3_, supplements prescribed for treatment in the US contain D_2_, while those for prevention, and all European supplements, contain D_3_ (7). VD from all sources is metabolized in the liver to 25-hydroxyvitamin D [25(OH)D] which is further transformed in the kidneys by the enzyme 1α-hydroxylase [1α(OH)ase, CYP27B1] to its active form, 1,25-dihydroxyvitamin D [1,25(OH)_2_D] (6). In addition, synthesis of the biologically active metabolite 1,25(OH)_2_D takes place intracellularly (8). The effects of 1,25(OH)_2_D are mediated through specific high-affinity vitamin D receptor (VDR) via upregulating or downregulating target genes (9).

### Local Immunomodulatory and Antiviral Activity of Vitamin D

During a lower respiratory infection, various factors, virus-dependent and host-dependent, regulate the development and severity of infections. It has been suggested that host reactions to viral infection, rather than the direct viral injury, are responsible for the clinical and pathological manifestations (10) and contribute to the development of RW after repeated ARTI. Different respiratory viruses will produce an immune host response mediated by both T and B cells.

In addition to well-researched functions in calcium homeostasis, VD and VDR modulate both the innate and the adaptive immune response, and play a key role in the balance between T-helper 1 and T-helper 2 (Th1-Th2) cytokines (11, 12).

*In vitro* studies suggest that VD induces a shift in the balance between Th1-type and Th2-type cytokines toward Th2 dominance (2). It has been shown that VD decreases the proinflammatory type 1 cytokines: IL-12, interferon-gamma (IFN-γ), IL-6, IL-8, tumor necrosis factor alpha (TNFα) and IL-17 and increase anti-inflammatory IL-10 and Th2 cytokines: IL-4 and IL-5 (13–15). A few human studies demonstrated the shift toward Th2, while others do not confirm these results. The relationship between serum VD levels and asthma remains controversial, and a U-shaped association has been suggested, with both VD deficiency and high levels of VD leading to a risk of asthma and allergy (16).

VD modulates B cell activities, influencing production of immunoglobulin E (IgE), and decreasing cell proliferation and differentiation but increasing apoptosis (15). In a British birth cohort, at the age of 45 years, IgE concentrations were higher in both subjects with VD <25 nmol/l and those with VD >135 nmol/l, suggesting that both low and high VD levels are associated with elevated IgE levels, confirming the U-shaped relationship (17). A review of *in vitro* experiments investigating the immunomodulatory activity of VD revealed no influence on replication or clearance of respiratory viruses in human respiratory epithelial cells (15).

The respiratory viruses, rhinovirus (RV) and respiratory syncytial virus (RSV) are reported to downregulate VDR mRNA expression in primary bronchial epithelial cells (PBECs). RV replication and its capacity to infect epithelial cells were found reduced in VD treated PBECs, suggesting that this might contribute to the antiviral activity of vitamin D (18).

*In vitro* and animal studies have shown an inhibitory effect of VD on airway smooth muscle cells, suggesting implication of VD in airway remodeling, which may be a primary event in asthma pathogenesis (19, 20).

**Figure 1:**
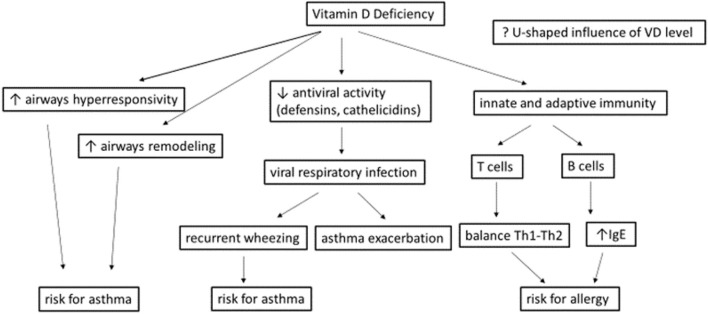
Potential effects of vitamins D deficiency during a viral respiratory infection [adapted from Mirzakhani et al. (22)].

Despite lack of solid documentation, it is generally accepted that VD deficiency has effects not only on calcium homeostasis and bone health but also on non-skeletal diseases (21), as shown in Figure 1 (22).

### Vitamin D Requirements in Childhood

Needs for VD intake depend on latitude, season, ethnicity, age, body weight, health status, dress habits and use of sunscreen creams. Many experts suggest that both children and adults should take ≥800–1,000 IU vitamin D/day from dietary and supplemental sources when exposure to sunlight is unable to provide it (23). Sacheck and colleagues showed that children at risk for vitamin D deficiency achieved a higher mean 25(OH)D serum level after 3 months daily supplementation with 2,000 IU/day than with 600 and 1,000 IU/day, doses closer to the current recommended daily allowance (24). The Endocrine Society Practice Guidelines Committee (Table 1) and the European Academy of Pediatrics (EAP) recommend for prevention in infants (0–1 year) and children (1–18 years) at risk of vitamin D deficiency, 400–1,000 IU/day, and 600–1,000 IU/day, respectively (25).

**Table 1:** Vitamin D requirements according to age; doses recommended by the Endocrine Society Practice Guidelines Committee, for prevention and treatment of vitamin D deficiency, and upper limits for administration without risk of adverse events (26).

**Recommended daily dose (IU) of vitamin D**	**0–6 months**	**6–12 months**	**1–3 years**	**4–8 years**	**8–18 years**
Prevention^*^	400–1,000		600–1,000		
Treatment of deficiency^**^	2,000		2,000		
Tolerated upper limit	2,000	2,000	4,000	4,000	4,000

*25(OH)D = 25 hydroxy vitamin D*.

* *In patients at risk for vitamin D deficiency*.

** *For at least 6 weeks, to achieve a blood level of 25(OH)D > 30 ng/ml*.

For treatment of deficiency, 2,000 IU/day for at least 6 weeks, to achieve a serum 25(OH)D level >30 ng/ml has been suggested, followed by doses as for prevention (26). Administration of VD to patients with low baseline 25(OH)D levels should be in doses sufficient to achieve normal levels (i.e., treatment of deficiency not supplementation). Studies to date have produced conflicting findings of relative efficacy but both vitamin D_2_ and D_3_ are recommended (26).

Few pediatricians, however, apply these guidelines in practice. DelGiudice and colleagues report that although most primary care providers are aware that vitamin D deficiency is common, fewer than half currently recommend 600–1,000 IU supplementation for their pediatric patients (27).

### Vitamin D Status: Deficiency—Insufficiency Definitions

Serum or plasma level of total 25(OH)D is currently used as the indicator of VD status (28). Almost all professional associations define VD status as sufficient when the level of 25(OH)D is at least 30 ng/mL, insufficient at 21–29 ng/mL and deficient at <20 ng/mL (7). The European Society for Pediatric Gastrenterology, Hepatology and Nutrition (ESPGAHN) characterizes as sufficient serum 25(OH)D concentrations >50 nmol/L (20 ng/ml), and <25 nmol/L (10 ng/ml) as severe deficiency (29). To convert 25(OH)D from ng/mL to nmol/L, multiply by 2.496 (28).

Current recommendations for VD screening concern only groups of children at risk for VD deficiency. Obesity, pigmented skin, inadequate diet intake, indoor lifestyle, lack of sunlight exposure (beyond a latitude of 35°), use of sun screen, liver disease, drugs (rifampicin, glucocorticoids, anticonvulsants) are all risk factors for VD deficiency and insufficiency in children (30). Following improved understanding of VD involvement in host reactions against infection and within the immune system, both innate and adaptive, the relevant guidelines have begun to include groups with recurrent RTI and asthma (31).

## Vitamin D Status and Recurrent Wheezing/Viral Respiratory Infection

Epidemiological and observational studies have demonstrated a clear association between VD deficiency and viral respiratory infections in certain contexts, while interventional studies on VD supplementation and/or VD status have had mixed findings.

### Relationship Between Vitamin D Deficiency and Wheezing and/or Viral Respiratory Tract Infections

The most common viruses responsible for acute respiratory infections in infants and children are the influenza virus, RVs, RSV and metapneumovirus. Only few VD studies have identified the exact type of viral infection, but found no association of VD levels with the presence of a certain virus, except in patients with positive RSV, RV or coinfections (32).

Eroglu and colleagues recently showed that 25(OH)D_3_ levels were significantly lower in children with RW than in a healthy control group, and had no relationship with hospitalization, oxygen, or steroid treatment (33). In an earlier study, a significantly lower mean 25(OH)D level was observed in patients with RW than in a healthy control group, and the level was negatively correlated with the duration of wheezing, number of wheezing episodes and systemic glucocorticoid need (34). Conversely, Pecanha and colleagues showed no association between 25(OH)D concentration and exacerbations, as assessed on the basis of hospitalizations, emergency department (ED) visits, and oral corticosteroid use in children with RW, but observed an association with onset of wheezing before the age of 1 year (35).

McNally and colleagues investigated 105 children aged <5 years with ARTI (bronchiolitis and pneumonia) requiring hospitalization, and 92 control children. Mean 25(OH)D level were not significantly different in the control and ARTI groups but were significantly lower in the 16 children with ARTI requiring PICU admission (15%) than in both the control subjects and children in the general pediatric ward. The authors concluded that deficient VD status may influence the severity of ARTI, but not the risk of hospitalization (36). Similarly, in a 17-center prospective cohort study of infants hospitalized with bronchiolitis, those with total 25(OH)D <20 ng/ml had an increased risk of intensive care and longer hospital length-of-stay (37). In a study comparing 64 infants hospitalized in a general pediatric ward for ARTI and 65 control subjects, all aged 1 month−2 years, mean 25(OH)D and prevalence of VD deficiency/insufficiency were similar in the two groups, and not associated with risk of hospitalization for ARTI (38). A review of 12 studies showed that the children with LRTI had significantly lower mean VD levels than the control subjects, and demonstrated a correlation between VD levels and the incidence and severity of LRTI (39).

Evidence generated from recent meta-analyses revealed that increased prenatal exposure to 25(OH)D (measured as cord blood or maternal venous blood) was inversely associated with risk for wheeze and/or RTI, in the offspring, while for asthma the evidence was mixed (40–42). The inverse association for wheeze was more pronounced and statistically significant in the studies that measured 25(OH)D levels in cord blood (43).

### Role of Vitamin D Supplementation and Wheezing (Antenatal—Postnatal, Prevention –Treatment)

Almost all interventional studies support the value of VD supplementation in pregnancy in reducing the prevalence of RW in infants. The findings of efficacy studies of VD supplementation in infancy are contradictory regarding viral respiratory infection.

#### Antenatal Vitamin D Supplementation

The VD Antenatal Asthma Reduction Trial (VDAART) was a trial of prenatal VD supplementation, in which 440 women were randomized to receive 4,000 IU/day VD, and 436 women only 400 IU. The incidence of asthma and RW in their children at the age of 3 years was lower by 6.1% in the group with the higher intake, but this did difference was not statistically significant (28). Prenatal VD supplementation did not affect the development of asthma and RW at the age of 6 years (44). These results suggest that antenatal supplementation alone does not provide adequate protection, and postnatal supplementation is needed. In a meta-analysis of data from 16 birth cohorts, Feng showed that increased antenatal exposure to 25(OH)D is inversely related to the risk of asthma and wheeze in the offspring, but not respiratory tract infections (43).

#### Postnatal Vitamin D Supplementation

In 703 healthy children aged 1–5 years, administration of 2,000 IU, compared with 400 IU, of VD for a minimum of 4 months between September and May did not reduce the prevalence of upper ARTI during the winter (45). A secondary analysis of this multisite RCT assessing whether wintertime high-dose VD supplementation reduces URTI symptom severity and frequency of ED visits compared with low-dose found no differences; no data about the baseline VD levels are available (46). Even in children with a baseline serum 25(OH)D level <30 ng/mL, the incidence per person-year was no different (47). Laboratory confirmed ARTI was slightly more frequent in the high-dose than the standard dose group, and the authors concluded that VD in doses >400 IU/day may not be indicated for preventing winter ARTIs in children (45). A 2017 meta-analysis of data from randomized controlled trials (RCTs) demonstrated that VD supplementation reduced the risk of at least one ARTI. Daily or weekly supplementation without additional bolus doses protected against ARTI, with the strongest effect in those with the lowest baseline 25(OH)D (48). Among 277 black infants born premature, 147 were consuming 200 IU/day from diet and 150 additional 400 IU/day from medical supplementation, which reduce the risk of RW by 12 months (49). Recently, 650 healthy children and adolescents were randomly assigned to taking 14,000 UI vitamin D weekly and 650 to taking placebo, for 8 months. No significant difference was observed between the vitamin D and placebo groups for influenza, but non-influenza respiratory viral infections were significantly reduced in the vitamin D group. When considering all respiratory virus infections, including influenza, the effect of vitamin D in reducing infection was also significant (50). In a recent prospective birth cohort study from China, infants receiving 400–600 IU of vitamin D from birth were divided into 4 groups, according to the average frequency of supplementation (0, 1–2, 3–4, and 5–7 days/week). Inverse trends were observed between supplementation and risk of ARTI, LRTI, and ARTI-related hospitalization (51).

Overall, the RCTs to date, while not uniform in their results, provide indications that VD supplementation, taken daily or weekly without bolus, can lower the risk of severe ARTI in children with low baseline 25(OH)D (2).

There is a lack of evidence on administration of VD to infants during an ARTI, and the optimal dose and timing. A recent Cochrane review evaluated 4 studies involving 780 children with pneumonia and 3 studies including 749 children with severe or very severe pneumonia, all aged <5 years. Various doses and methods of administration of VD were used, but, because of low and very low-quality evidence, the reviewers remained uncertain as to whether oral vitamin D as an a adjunct to treatment of acute pneumonia in children <5 years has an effect on outcome (52).

### Vitamin D and Primary Prevention of Respiratory Allergies

RW caused by respiratory viral and/or bacterial infections in infancy and early childhood often persists after the age of 7 years, and asthma becomes established. Interventional studies in pregnancy tend to support the value of maternal VD supplementation in reducing the prevalence of childhood asthma, in contrast to studies on supplementation in infancy, which show contradictory results. Once initiated, asthma appears to have an association with VD status. A systematic review identified 23 manuscripts (two case-control, 12 cohort and nine cross-sectional studies) and found that higher serum levels of VD are associated with a reduced risk of asthma exacerbations, but little evidence to suggest an association with asthma incidence, prevalence or severity (53).

## Vitamin D As Biomarker

In local inflammation produced by respiratory viruses, the activity of enzymes responsible for synthesis [1α(OH)ase] and degradation [24(OH)ase] of the active vitamin D metabolite 1,25(OH)D_2_ is dysregulated. In order to exercise their antiviral role, local macrophage and other immune cells release antiviral proteins (cathelicidin, defensis and innate interferons) (9). It is possible that in more severe viral infections the higher release of antiviral proteins decreases the activity of inactivating 24(OH)ase and increases the activity of 1α(OH)ase. Consequently, the concentration of 1,25(OH)D_2_ is increased in extra-renal tissues, to the detriment of serum 25(OH)D from which it is synthesized. A severe viral infection during periods when VD intake and exposure to sunlight are reduced may thus result in low serum 25(OH)D. It has yet to be shown in the real-life situation whether or not VD level could be used as a biomarker for the severity of ARTIs. Current evidence suggests that VD supplementation may reduce the risk of severe ARTI in some clinical situations, depending on both host and virus characteristics. The changes at the molecular level may differ, depending on type of virus. Urashima and colleagues, studying the effect of 1,200 IU/day of vitamin D supplementation vs. placebo on the incidence of seasonal influenza A and B among children aged 6–15 years, showed reduced incidence of influenza A but not of influenza B infection (54). Production of active metabolite VD resulted in initial increase in 25(OH)D in blood, and consequently in the respiratory tract; sufficient VD increases the antiviral activity of respiratory epithelial cells (18).

As VD supplementation is not followed by reduction in RW or asthma prevalence, although VD deficiency is associated with both conditions, an inverse causal relationship could be hypothesized. In both asthma and in RW, local 25(OH)D is needed to produce 1,2(OH)_2_D. Recent studies showed that many innate immune cells can synthesize 1,2(OH)_2_D from 25(OH)D (9). Whether low VD during ARTI is a co-factor in their appearance, or a result of local transformation to the active form to combat infection is unclear. Sophisticated determinations to conclude which children respond positively to intervention are not feasible in primary care, but it may be useful to measure VD during a new ARTI, and to correct deficiency, in children with RW.

## Conclusions

New biomarkers are needed for early identification of those children with RW who are at increased risk of severe ARTI and require intensive treatment and close follow-up. VD status appears to be a suitable candidate in certain populations. As VD deficiency could be a risk factor for causing RW in children, measurement of their serum level of 25(OH)D is recommended. In the case of deficiency, VD should be administrated in doses according to age for at least 6 weeks, until achievement of normal blood 25(OH)D level, followed by supplementation when exposure to sunlight is inadequate. Higher doses of VD in this group in an effort to influence the clinical course and prevent asthma appear to be of no additional benefit. In a new era of personalized medicine, decisions based on appropriate treatment for different wheezing phenotypes may be possible.

## Further Research

Further research is needed in this area, including well-designed RCT or longitudinal study, to confirm VD role in severe infection and analyze the risk factor in recurrent wheezing.

## Author Contributions

GF and CB had the conception, designed the work, and collected the data. GF, CB, and LS contributed to the data analysis, its interpretation and to the article's writing and editing. AB and MZ made the critical revision of the article. All authors contributed to the article and approved the submitted version.

## Conflict of Interest

The authors declare that the research was conducted in the absence of any commercial or financial relationships that could be construed as a potential conflict of interest.
